# Transcriptional analysis of micronutrient zinc-associated response for enhanced carbohydrate utilization and earlier solventogenesis in *Clostridium acetobutylicum*

**DOI:** 10.1038/srep16598

**Published:** 2015-11-20

**Authors:** You-Duo Wu, Chuang Xue, Li-Jie Chen, Hui-Hui Wan, Feng-Wu Bai

**Affiliations:** 1 School of Life Science and Biotechnology, Dalian University of Technology, Dalian 116024, China; 2 State Key Laboratory of Fine Chemicals, Dalian University of Technology, Dalian 116024, Liaoning, China; 3 School of Life Sciences and Biotechnology, Shanghai Jiao Tong University, Shanghai 200240, China

## Abstract

The micronutrient zinc plays vital roles in ABE fermentation by *Clostridium acetobutylicum*. In order to elucidate the zinc-associated response for enhanced glucose utilization and earlier solventogenesis, transcriptional analysis was performed on cells grown in glucose medium at the exponential growth phase of 16 h without/with supplementary zinc. Correspondingly, the gene *glcG* (CAC0570) encoding a glucose-specific PTS was significantly upregulated accompanied with the other two genes CAC1353 and CAC1354 for glucose transport in the presence of zinc. Additionally, genes involved in the metabolisms of six other carbohydrates (maltose, cellobiose, fructose, mannose, xylose and arabinose) were differentially expressed, indicating that the regulatory effect of micronutrient zinc is carbohydrate-specific with respects to the improved/inhibited carbohydrate utilization. More importantly, multiple genes responsible for glycolysis (*glcK* and *pykA*), acidogenesis (*thlA, crt, etfA, etfB* and *bcd*) and solventogenesis (*ctfB* and *bdhA*) of *C. acetobutylicum* prominently responded to the supplementary zinc at differential expression levels. Comparative analysis of intracellular metabolites revealed that the branch node intermediates such as acetyl-CoA, acetoacetyl-CoA, butyl-CoA, and reducing power NADH remained relatively lower whereas more ATP was generated due to enhanced glycolysis pathway and earlier initiation of solventogenesis, suggesting that the micronutrient zinc-associated response for the selected intracellular metabolisms is significantly pleiotropic.

Biobutanol produced via biphasic ABE fermentation by the solventogenic clostridia has attracted worldwide attention due to its remarkable advantages as a promising and renewable biofuel^1^,^2^. In particular, the spore-forming, strict anaerobic *Clostridium acetobutylicum* is an important ABE-producing organism with tremendous superiorities in terms of fermentative feedstock diversity and flexibility, biosynthetic capability and stress tolerance^3^,^4^. *C. acetobutylicum* is capable of fermenting a wide range of simple and complex carbohydrates, including polysaccharides, disaccharides and monosaccharides (hexose and pentose), making the ABE fermentation more flexible in the feedstock selection^5^,^6^,^7^,^8^.

Since the sequencing of model strain *C. acetobutylicum* ATCC 824 has been accomplished and successfully annotated^9^, several transcriptomic studies using DNA microarrays and RNA-seq have been published in the recent years aiming to unveil the transcriptional program associated with carbohydrate utilization^10^,^11^, biphasic metabolism^12^,^13^,^14^, metabolite stress response^15^,^16^,^17^,^18^, sporulation^19^,^20^ and physiological changes^21^,^22^. A great deal of detailed transcriptional data has been used for identification and characterization of responsive genes network and regulatory circuitry^23^,^24^,^25^. For example, differential carbohydrate utilization involved numerous genes in complicated metabolic and regulatory network associated with carbon catabolite repression (CCR) as well as metabolite stress responses^11^,^16^. Additionally, microbial ABE biosynthesis will inevitably depend on the regulatory response of the strain to respective carbohydrate and nutrient availability in the medium, which is still incompletely understood at the molecular level. Therefore, understanding these complex physiology and regulatory mechanisms underlying specific response at the systems level is a fundamental requirement for the exploration of potential target genes and thus the development of genetic and metabolic engineered strains^18^.

According to previous studies, zinc is an important micronutrient for cell growth and cellular metabolism of almost all living organisms, acting as not only the cofactor of a myriad of enzymes such as alcohol dehydrogenase, but also the structural component of proteins including some ribosomal proteins and zinc finger proteins^26^. It has been reported that zinc supplementation in the medium was beneficial for ethanol production^26^,^27^. As a matter of fact, approximately 3% of the *Saccharomyces cerevisiae* proteome function requires zinc and a total of 105 proteins in *S. cerevisiae* have been identified using zinc as a cofactor^27^,^28^. In addition, zinc is also required by 360 proteins in *S. cerevisiae* that have Zn-binding domains for maintaining their structural stability^27^. More importantly, zinc finger structures represent the diverse superfamily of nucleic acid binding proteins and play critical roles in transcriptional regulation of cellular metabolic network^29^.

Until recently, zinc has been proven to be significantly involved in ABE fermentation by *C. acetobutylicum* with respects to enhanced glucose utilization, cell growth, acids re-assimilation and butanol biosynthesis as well as earlier initiation of solventogenesis^30^. However, little is known about how zinc acts upon the physiology and genetics of *C. acetobutylicum* at the molecular level. Therefore, in-depth knowledge of the micronutrient zinc-associated response for these selected metabolisms is thus essential for the enrichment of regulatory mechanism existing in *C. acetobutylicum* and development of large-scale ABE fermentation.

## Results and Discussion

### Transcriptional analysis of genes for glucose-specific PTS

According to our previous study^30^, micronutrient zinc at milligram level could contribute to not only rapid glucose utilization, enhanced cell growth, acids re-assimilation and butanol production but also earlier initiation of solventogenesis by *C. acetobutylicum*. During the fermentation using 7% glucose as sole carbon source, for example, at the exponential growth phase of 16 h, the specific glucose consumption rate of 1.00 g/g-DCW/h was dramatically achieved compared to that of 0.55 g/g-DCW/h in the control without zinc supplementation. Both acetate and butyrate were dramatically decreased by 25% and 44% respectively in the presence of zinc while no significant difference in acids levels was observed at 8 h or 12 h of fermentation. Strikingly, as high as ~6.5 g/L butanol and ~10.5 g/L ABE were produced efficiently with ~30 g/L glucose utilized while only ~3.5 g/L butanol and ~6.5 g/L ABE produced with ~20 g/L glucose utilized in the control, suggesting the solventogenesis was prominently initiated by supplementary zinc at this time point. Therefore, cells grown in the glucose medium without/with supplementary zinc at the exponential growth phase of 16 h were used for target total cellular RNA isolation and transcriptional analysis with RNA-Seq to elucidate the zinc-associated response for not only enhanced glucose utilization, acid re-assimilation but also earlier initiation of solventogenesis by *C. acetobutylicum*. Correspondingly, the data obtained from transcriptional analysis showed that three genes for two glucose PTS were significantly influenced by supplementary zinc. The expression of gene *glcG* (CAC0570), typically belonging to the *Glc* family and encoding glucose PTS IICBA, was 3.62-fold upregulated. Additionally, the expression of the other two genes CAC1353 and CAC1354 encoding the putative glucose PTS were upregulated by 1.76- and 1.77-fold, respectively. Based on the Q-RT-PCR verification, similar expression patterns of these three genes (3.82-, 2.05- and 2.33-fold upregulation respectively) were observed at 16 h of fermentation with 0.0005 g/L ZnSO_4_.7H_2_O supplementation, suggesting zinc convincingly acted upon the glucose PTS at the transcriptional level.

In nature, the gene *glcG* was well investigated with respects to its characterization and transport mechanism in *C. acetobutylicum*, which appeared to be associated with a BglG-type regulator^31^. As previously documented, *glcG* was induced by glucose, maltose and starch while neither CAC1353 nor CAC1354 had significantly altered gene expression using 11 different carbohydrate tested^10^. Interestingly, it has been also reported that no effect on cell growth or glucose utilization was observed while co-utilization of xylose and arabinose was achieved in the presence of glucose after *glcG* was inactivated^32^, implying that the *glcG* inactivation might play a significant role in alleviating CCR rather than facilitating glucose utilization. Li *et al.*^33^ hypothesized that the internal glucose 6-phosphate concentration might be reduced by *glcG* inactivation, which in turn affected xylose utilization by triggering an HPr S46-phosphate-independent ccpA-cre interaction, or by acting as an anti-inducer for the xylose repressor. Alsaker *et al.*^16^ found that, with 45 mM acetate and 30 mM butyrate stress respectively, *glcG* was highly expressed 10 min post-stress, and then differentially downregulated later. In our study, *glcG* was also significantly upregulated by 4.93-fold only in the presence of zinc based on Q-RT-PCR verification at the early exponential growth phase of 8 h, whereas no apparent difference in acids and butanol levels was detected without/with zinc supplementation, suggesting zinc was apparently involved in transcriptional regulation of *glcG* in *C. acetobutylicum*. Also, genes CAC1353 and CAC1354 were upregulated by 1.46- and 1.94-fold at this time point. Together with the downregulated expression (approximately 0.3 ~ 0.5-fold) for all these three genes at 32 h via Q-RT-PCR verification, potentially coordinated action of multiple genes or cellular subsystems involved the biophysical, metabolic, or regulatory ensembles. Nevertheless, little was known about the importance of both CAC1353 and CAC1354 on glucose utilization, and our RNA-Seq data provoked further studies on detailed zinc-associated action upon these two PTS.

### Carbohydrate-specific effect of micronutrient zinc on ABE fermentation

**Figure 1:**
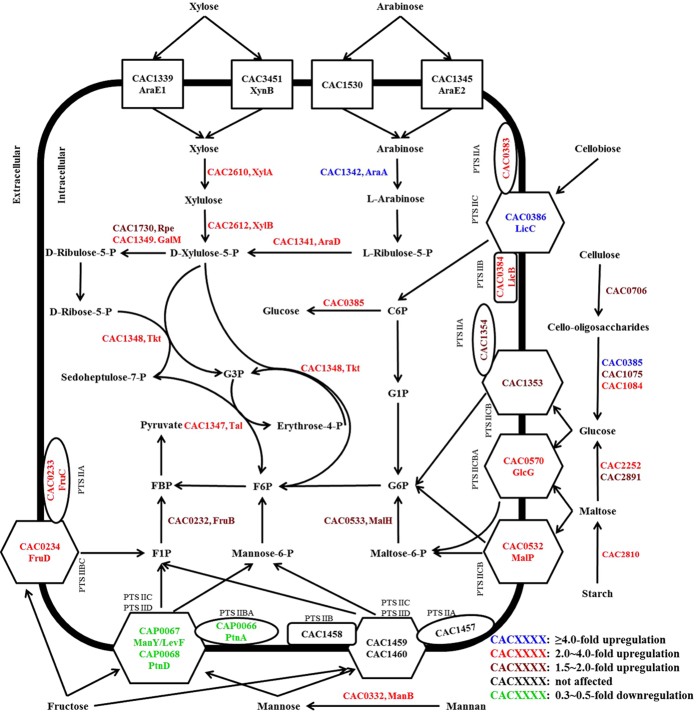
Overview of selected processes and related genes responsible for polysaccharides, disaccharides and monosaccharides (hexose and pentose) utilization of *C. acetobutylicum*. Expression ratios (zinc/control) of selected genes were ≥1.50 for upregulation and ≤0.50 for downregulation. ORF numbers, gene names and protein functions are based on Nölling *et al.*^9^.

**Figure 2:**
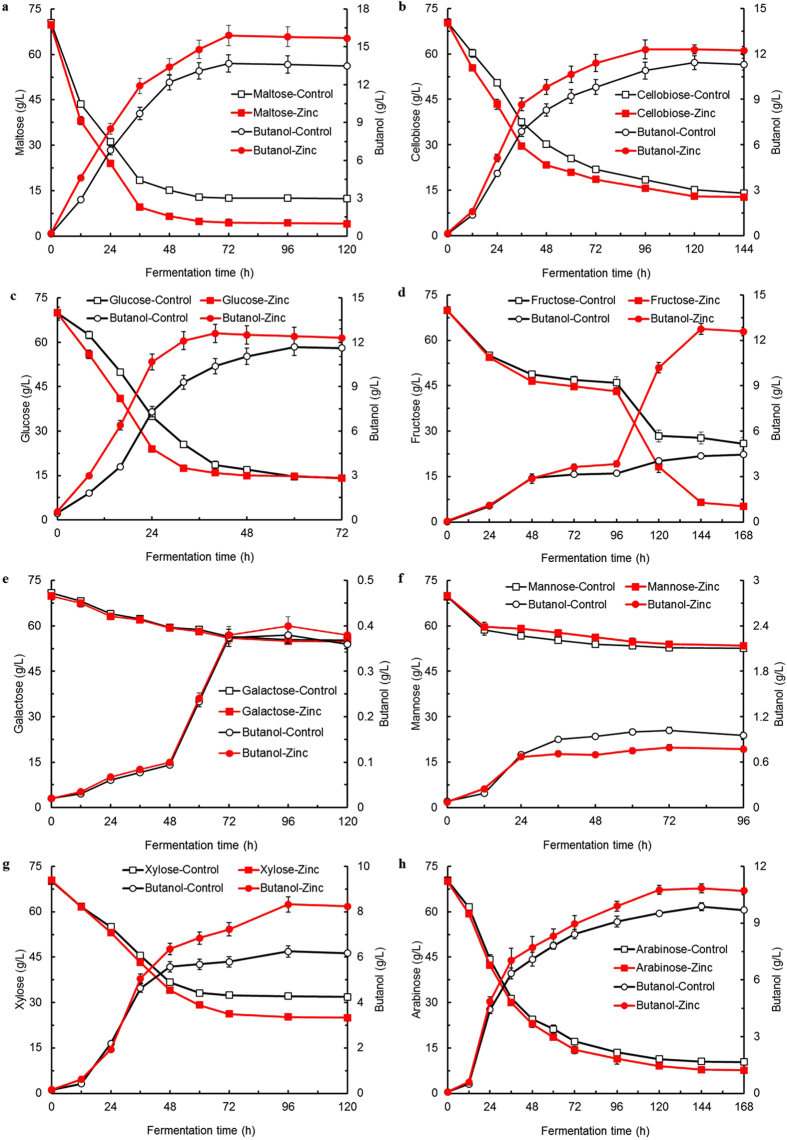
Comparative performance of batch ABE fermentation by *C. acetobutylicum* using differential carbohydrate as sole carbon source respectively without/with supplementary zinc. Residual carbohydrate and butanol production from each of (**a**) Maltose; (**b**) Cellobiose; (**c**) Glucose; (**d**) Fructose; (**e**) Galactose; (**f**) Mannose; (**g**) Xylose; (**h**) Arabinose.

In addition to glucose, multiple genes responsible for the utilization of other carbohydrates including two disaccharides (maltose and cellobiose), two hexoses (fructose and mannose) and two pentoses (xylose and arabinose) were differentially expressed by supplementary zinc as illustrated in Fig. 1 (detailed genes information shown in Supplementary Table S1 online), implying potentially regulatory effect of zinc on the utilization of these carbohydrates. In order to fully elucidate the zinc-associated response for the physiological behavior of *C. acetobutylicum*, batch ABE fermentation was further performed using carbohydrates above as sole carbon source respectively without/with supplementary zinc. The fermentation results were shown in Fig. 2 and Table 1 (data in each table cell shown as without/with zinc supplementation). For example, supplementary zinc led to as high as 15.9 g/L butanol produced from 65.5 g/L maltose compared to those of 13.7 g/L and 58.0 g/L obtained in the control (see Fig. 2a). On the basis of the *C. acetobutylicum* genome sequence analysis^9^, as for disaccharides, the expression of genes *malP* (CAC0532) and *malH* (CAC0533), located in an operon for a putative maltose-specific PTS IICB and a maltose-6′-phosphate glucosidase, respectively, were 2.66-fold and 1.51-fold upregulated. Additionally, the expression of another two putative α-glucosidase genes CAC2252, CAC2891 and a glucoamylase gene CAC2810 were differentially upregulated by 2.87-, 1.79- and 2.03-fold, respectively. It should be noted that these three genes were all strongly induced by starch and maltose compared to glucose and thus represented higher expression levels^10^. According to a recent study, two α-glucosidase genes (CAC2252 and CAC2891) were cloned from *C. acetobutylicum* ATCC 824 and separately expressed in *C. tyrobutyricum* together with gene *adhE2* (CAP0035). The mutants were evaluated for their abilities to use maltose and soluble starch as substrates in batch fermentations and produced more butanol (17.2 vs 11.2 g/L) from maltose compared to *C. acetobutylicum* ATCC 824^34^. Furthermore, the genes for the cellobiose-specific PTS, located in an operon and encoding a PTS IIA (CAC0383), a PTS IIB (CAC0384), a β-glucosidase (CAC0385) and a PTS IIC (CAC0386), were highly expressed by 3.20 ~ 5.19-fold whereas the expression of this operon was induced more than 6-fold by cellobiose compared to other carbohydrates as reported before^10^. As a consequence, cellobiose was efficiently utilized along with enhanced ABE biosynthetic capabilities in the presence of zinc (see Fig. 2b). These results suggested that zinc might play a role as an inducing factor involved in maltose/cellobiose utilization of *C. acetobutylicum*. Similarly, zinc also resulted in earlier solventogenesis coupled with less acids accumulation during the ABE fermentation.

On the other hand, it was proposed that there was very little difference in gene expression levels when cells were grown on glucose, fructose or mannose, respectively, indicating that fructose/mannose might be the preferred carbohydrates for *C. acetobutylicum*^10^. Unexpectedly, compared to glucose (see Fig. 2c), rather insufficient utilization of fructose/mannose was observed as well as poor ABE production due to earlier termination of fermentation without solventogenesis in the absence of zinc. Strikingly, the fructose utilization was restored with apparent solventogenesis initiation after zinc was supplemented into the medium (see Fig. 2d). Correspondingly, two types of PTS for fructose and mannose uptake were also prominently influenced. One fructose-specific PTS is encoded by *fru* operon that encompasses genes (CAC0231–CAC0234) for a putative DeoR-type transcriptional regulator (*fruR*), a 1-phosphofructokinase (*fruB*), a PTS IIA (*fruC*) and a PTS IIBC (*fruD*). It has been previously reported that the *fru* operon was proven to be significantly involved in the regulation of fructose uptake in *C. acetobutylicum*^35^. In response, all the genes in this operon were differentially upregulated by 1.51 ~ 2.76-fold, which potentially contributed to the improved fructose utilization of 63.5 g/L and restored soventogenesis with butanol production of 12.8 g/L. Interestingly, as for galactose illustrated in Fig. 2e, little difference was observed in terms of galactose utilization and butanol production without/with zinc supplementation, which was well consistent with unaffected expression levels of genes involved in galactose transport or metabolism based on our RNA-Seq data. On the contrary, genes (CAP0066–CAP0068) for the other PTS involved in fructose/mannose uptake were strongly downregulated by 0.33 ~ 0.45-fold while the second fructose/mannose PTS encoded by CAC1457–CAC1460 represented no significant difference on the expression levels in the presence of zinc, which might account for the lower mannose utilization and butanol production of only 16.4 g/L and 0.8 g/L compared to those of the control (see Fig. 2f). Additionally, the cell growth was affected to some extent with a maximum OD_620_ of 2.1 compared to that of 2.3 obtained in the control.

More importantly, significant improvements on xylose utilization and butanol production were observed while arabinose utilization was increased to a lesser extent after zinc was supplemented into the medium (see Fig. 2g,h). With respect to the genes involved in pentose metabolism, five genes (CAC1341, CAC1342, CAC1347–CAC1349), located in the region of the genome encompassing CAC1339–CAC1349 responsible for arabinose/xylose metabolism, were differentially upregulated in response to the zinc supplementation. Among them, genes CAC1341 and CAC1342 of an operon encoding an L-ribulose-5-phosphate 4-epimerase (*araD*) and an L-arabinose isomerase (*araA*), respectively, offered 2.33- and 4.84-fold expression levels. Moreover, the expression of genes CAC1347–CAC1349, located in another operon encoding a transaldolase (*tal*), a transketolase (*tkt*) and an epimerase (*galM*), respectively, were elevated by 3.19-, 2.44- and 2.77-fold. Additionally, three other genes CAC1730, CAC2610 and CAC2612 encoding a ribulose-phosphate 3-epimerase (*rpe*), a xylose isomerase (*xylA*) and a xylulose kinase (*xylB*), were 1.52-, 2.29- and 2.31-fold upregulated. Despite more efforts to engineer xylose/arabinose (co)utilizing capabilities, the regulatory mechanisms remain not clear^36^,^37^,^38^. According to previous studies, the overexpression of *xylA* or *xylB* resulted in improved xylose utilization while the inactivation of *xylA* or *xylB* gene led to insufficient xylose utilization^32^,^39^, suggesting both *xylA* and *xylB* may be essential for xylose metabolism in *C. acetobutylicum*. Similarly, it has been reported that three genes *xylT, xylA,* and *xylB* from *C. acetobutylicum* were co-overexpressed with *adhE2* (CAP0035) in *C. tyrobutyrium* (△ack). The mutant was able to simultaneously consume glucose and xylose at comparable rates for butanol production up to 12.0 g/L^40^. In addition, overexpressing the gene *tal* from *E. coli* was able to improve xylose utilization and ABE production^41^, and combined overexpression of four genes *tal, tkl, rpe* and *rpi* (CAC2880) also led to a significantly improved xylose-utilizing capability and ABE production^36^. These results suggested that potential mechanisms responding to the supplementary zinc were also involved in xylose/arabinose metabolism of *C. acetobutylicum*.

It should be noted that the L-ribulose-5-phosphate 4-epimerase harbors a zinc ion binding site, implying a potentially regulatory interaction of zinc with this enzyme. In fact, it is well accepted that zinc can be sequestered as catalytic or structural components^27^. For example, the different binding of zinc with regulatory proteins could influence the glycolytic enzyme activities^27^. It was proposed previously that the enhanced ethanol production was ascribed to the increase in the activity of ethanol dehydrogenase using zinc as the cofactor^26^,^27^,^28^,^29^. Similarly, the activity of butanol dehydrogenase responsible for butanol biosysnthesis in *C. acetobutylicum* could be restored *in vivo* when zinc was supplemented into the buffer^42^, suggesting zinc was a key cofactor affecting the biological function of butanol dehydrogenase. Correspondingly, butanol was efficiently produced in the presence of zinc during batch ABE fermentation using glucose as sole carbon source. On the other hand, zinc is also the main component of the zinc finger structures, which are found in many microorganisms such as yeast and known to exert important roles in transcriptional regulation of cellular metabolic network^29^. For example, it has reported that overexpression of genes *Msn2, Msn4* and *Crz1* encoding zinc finger proteins as transcription factors could contribute to the improved ethanol tolerance and ethanol fermentation performance^43^,^44^. Until recently, two genes SMB_G1518-1519 (annotated as CAC1493 and CAC1494 in the genome of type strain *C. acetobutylicum* ATCC 824) involved in butanol tolerance were identified and characterized, which could encode proteins containing Zn-finger DNA-binding domain as transcriptional regulators. The inactivation of gene SMB_G1518, SMB_G1519 or both genes enabled the strains to grow faster and increased butanol tolerance upon butanol challenge^24^. Nevertheless, little is still known about how zinc finger proteins act upon the physiology of clostridia.

### Zinc-associated response to central carbon metabolism

**Figure 3:**
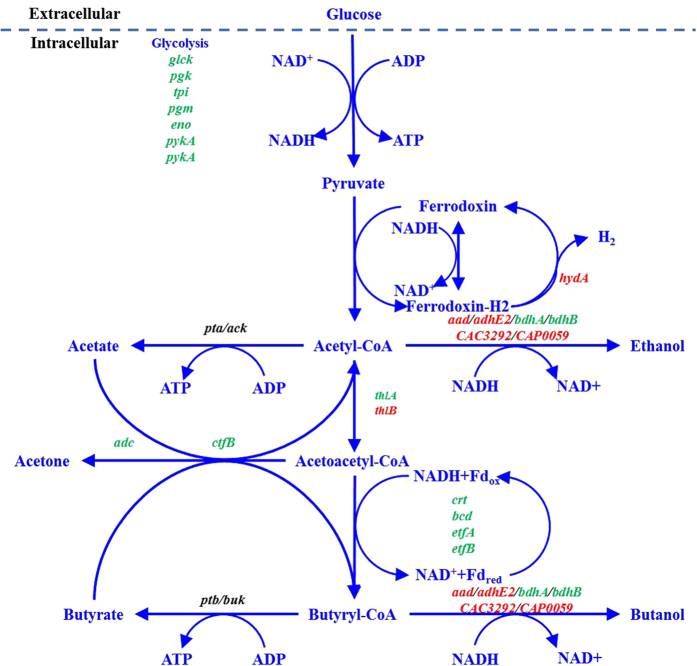
Comparative analysis of genes expression involved in primary metabolism of *C. acetobutylicum* using glucose as sole carbon source without/with supplementary zinc. With supplementary zinc, green written genes were upregulated, and red written genes were downregulated, whereas black written genes remained unaffected. ORF numbers, gene names and protein functions are based on Nölling *et al.*^9^.

**Figure 4:**
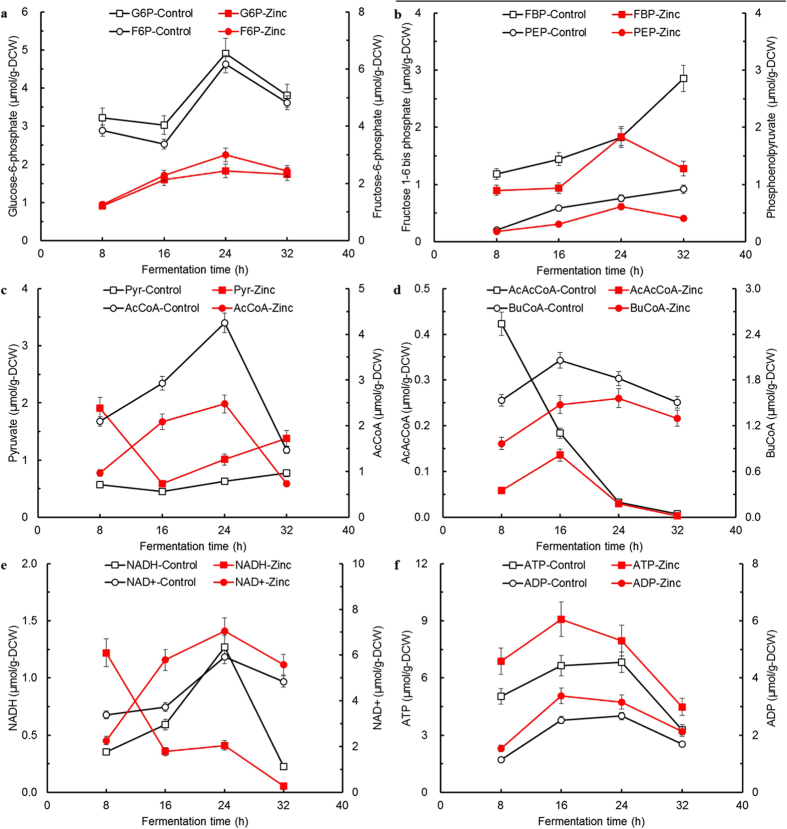
Comparative analysis of key intermediate metabolites involved in central carbon metabolism of *C. acetobutylicum* using glucose as sole carbon source without/with supplementary zinc.

When using glucose as sole carbon source, it is first phosphorylated to glucose-6-phosphate, and then subsequently metabolized through the Embden-Meyerhof-Parnas pathway (EMP) toward pyruvate. As shown in Fig. 3, comparative analysis of the primary metabolic genes expression and key intermediate metabolites was further performed (detailed genes information shown in Supplementary Table S2 online). Firstly, the glycolytic genes for converting glucose to pyruvate were slightly upregulated by no more than 1.25-fold. Among these genes, the first gene *glcK* (CAC2613) encoding a putative glucokinase was upregulated by 1.21-fold. Most of the remaining glycolytic genes CAC0710–CAC0713, CAC0518 and CAC1036 showed a little higher expression levels ranging from 1.15 ~ 1.20-fold. Additionally, no apparent difference on the expression levels of three genes CAC0517, CAC0709 and CAC0827 was observed. Given the importance of these genes, previous study showed that inhibition of glucose uptake was observed under stress condition which was consistent with the downregulation of both *glcG* and *glcK*^16^. Double-overexpression of both the gene *pfkA* (CAC0517) and *pykA* (CAC0518), encoding 6-phosphofructokinase and pyruvate kinase, respectively, could lead to enhanced butanol production and increased intracellular ATP as well as NADH levels^45^. Additionally, it has been reported that the gene *eno* (CAC0713) expression was strongly upregulated by butyrate and acetate while strongly downregulated by butanol. Another gene *pgm* (CAC0712) expression was upregulated by butyrate stress but was downregulated by 50 mM butanol stress^16^. In our study, much lower acids and higher butanol levels due to zinc supplementation were present in the growing environment at 16 h, leading to differentially upregulated expression of genes *eno, pgm* and *hydA* by supplementary zinc. This phenomenon suggested that zinc was convincingly involved in the transcriptional regulation of these selected genes in *C. acetobutylicum*. On the other hand, with respects to key intermediate metabolites involved in glycolysis at different sampling time (Fig. 4), glucose-6-phosphate (G6P), fructose-6-phosphate (F6P), fructose 1–6 bis phosphate (FBP) and phosphoenolpyruvate (PEP) were kept relatively lower levels within the first 32 h of fermentation rather than accumulated due to more glucose utilization under zinc supplementation condition (see Fig. 4a,b), suggesting that zinc could significantly lead to enhanced glycolysis towards acidogenesis or solventogenesis of *C. acetobutylicum*.

In terms of the expression of the pyruvate to butyryl-CoA formation genes, *thlA* (CAC2873), *crt* (CAC2712), *etfA* (CAC2709), *etfB* (CAC2710), and *bcd* (CAC2711) were differentially upregulated by no more than 1.75-fold. Surprisingly, *thlB* (CAC0078) encoding acetyl-CoA acetyltransferase was 0.65-fold downregulated compared to 1.46-fold upregulation of the primary thiolase gene *thlA* in the presence of zinc. As for the two thiolase encoding genes *thlA* (CAC2873) and *thlB* (CAP0078), only *thlA* is physiologically relevant whereas *thlB* is only briefly induced with relatively low expression levels during the transition from acidogenesis to solventogenesis and kept at steady levels^46^,^47^. Nevertheless, little was known about the *thlB* regulation on clostridial metabolisms. As a matter of fact, it’s well accepted that thiolase plays a key role in the production of both acids and solvents by catalyzing two molecules of acetyl-CoA into one acetoacetyl-CoA and controlling the carbon flow into these pathways. The thiolase activity is steadily increasing throughout the time course of fermentation with the maximum activity in the early stationary growth phase^48^. Accordingly, thiolase engineering has been demonstrated to be applicable for enhanced butanol production by *C. acetobutylicum*^49^. It should be noted that thiolase may also play an indirect role in acid re-assimilation^48^. Conceivably, the upregulation of these genes could support the hypothesis of an increased intracellular availability of carbon flux towards C_4_ compounds, i.e. butyrate and butanol as the main fermentation products, which was in accordance with the rapid butanol rather than butyrate production observed. Moreover, little difference was detected in terms of the expression of the acetate/butyrate biosynthesis genes *pta* (CAC1742), *ack* (CAC1743), *ptb* (CAC3076) and *buk* (CAC3075). As for the ATP mainly generated from the acidogenesis (especially butyrate-producing pathway), relatively higher level of ATP was detected (see Fig. 4f), which well corresponded to the maximum OD_620_ (~2.9 vs ~2.4) and appeared to contradictory with drastically decreased acids especially butyrate production (~1.0 vs ~1.8 g/L) compared to those of the control.

To our best knowledge, the intracellular redox equilibrium during acidogenesis is balanced by redirecting electrons into acetate/H_2_ or by oxidating NADH through butyrate accumulation (acetyl-CoA converted to butyl-CoA). During the subsequent solventogenesis, NADH is further shunted to ABE formation along with downregulated hydrogenase activity^50^. Correspondingly, expression of the hydrogenase gene *hydA* (CAC0028) is also downregulated during solventogenesis^19^. Alsaker *et al.*^16^ found that *hydA* was generally downregulated by both acetate and butyrate stresses. As can be seen in Fig. 4e, much higher level of intracellular NADH (1.22 vs 0.35 μmol/g-DCW) at 8 h of fermentation was detected in the presence of zinc, which was then rapidly decreased to 0.36 μmol/g-DCW at 16 h of fermentation coupled with slightly downregulated *hydA* expression to 0.86-fold compared to that in the control at the exponential growth phase of 16 h, indicating that earlier solventogenesis was prominently initiated. Given the importance of the solventogenic genes, the expression of *ctfB* (CAP0164) was 1.62-fold elevated while the other gene *ctfA* expression (CAP0163) was not detectable under both conditions. Furthermore, the expression of another gene *adc* (CAP0165) responsible for acetone formation and acids re-assimilation was 1.41-fold upregulated, which was consistent with the rapid acetone production (3.6 vs 2.4 g/L) observed^30^. In nature, *ctfB* is part of a tricistronic operon (*aad-ctfA-ctfB*) containing the gene *aad*, which encodes the alcohol/aldehyde dehydrogenase and is regarded as the primary gene for ethanol/butanol biosynthetic pathway^51^. It has been reported that the *ctfB* asRNA led to apparent downregulation of both *ctfB* and *aad*, thus in turn resulting in lower butanol production^52^ due to their existence in the same mRNA transcript. Unexpectedly, 0.61-fold downregulation of *aad* was detected, which appeared to be contradictory with the enhanced ethanol/butanol biosynthetic capability observed in this study. With respects to additional genes for alcohol-biosynthetic enzymes, including *adhE2* (CAP0035), CAP0059, CAC3292, *bdhB* (CAC3298) and *bdhA* (CAC3299)^42^,^53^,^54^, no difference was observed with respect to the *adhE2* expression levels under both conditions while the expression of two genes CAP0059 and CAC 3292 were drastically downregulated by 0.21- and 0.63-fold. Nevertheless, the expression of *bdhA* and *bdhB* were 1.08- and 1.35-fold upregulated, respectively, suggesting that zinc could play vital roles in regulating both gene expression and enzyme activity of butanol dehydrogenase^42^. Combined with the comparative analysis of another four intermediate metabolites involved in acidogenesis and solventogenesis (see Fig. 3c,d), pyruvate (Pyr) was accumulated relatively higher (1.91 vs 0.57 μmol/g-DCW) at 8 h of fermentation followed by rapid utilization while acetyl-CoA (AcCoA), acetoacetyl-CoA (AcAcCoA), and butyl-CoA (BuCoA) remained much lower levels within the first 32 h of fermentation compared to those detected in the control, respectively, indicating that the rapid ABE production was highly ascribed to the transcriptional regulation on glycolytic, acidogensic and solventogenic genes by supplementary zinc, which thus contributed to the earlier initiation of solventogenesis in *C. acetobutylicum*.

Until now, many studies have been reported about the metal-directed regulation on clostridia. For example, phosphofructokinase required both magnesium and ammonium or potassium. Magnesium forms a complex with ATP, which is perhaps the true substrate of the enzymatic reaction^55^. It has been suggested that ammonium may stimulate glycolysis through the activation of phosphofructokinase to increase the rate of ATP synthesis, which is necessary for nitrogen fixation^56^. The acetate kinase from different strains of clostridia had a strict requirement for magnesium and seemed to have a requirement for manganese^57^. Furthermore, phosphate acetyltransferase from *C. acetobutylicum*^58^ was activated by ammonium and potassium and inhibited by sodium. Iron was an indispensable cofactor involved in four iron–sulfur clusters for the active site of clostridial [FeFe]-hydrogenase, which catalyzes the reduction of protons to yield hydrogen^59^. Additionally, both NADH- and NADPH-dependent butanol dehydrogenase from crude extract required zinc in the buffer to obtain a higher recovery of activities during the purification^42^. Recently, proteomic analysis revealed the broader effects of calcium at the cellular and protein levels on carbohydrate utilization, cell growth, acids re-assimilation, butanol production and tolerance in ABE fermentation by *C. beijerinckii* NCIMB 8052^60^. More importantly, Han *et al.*^60^ observed 2.3-, 1.2-, and 1.4-fold increases in activity for CoA-transferase, acetate kinase, and acetoacetate decarboxylase, respectively, while a negligible difference in activity for butanol dehydrogenase and butyraldehyde dehydrogenase when calcium was supplemented into the assay mixtures. Nevertheless, these metal-driven stimulatory effects on ABE fermentation were somehow multifactorial and multifunctional due to the complicated metabolic network of clostridia. Similarly, the results above demonstrated that the micronutrient zinc could also play crucial roles facilitating the efficiency of clostridial fermentation and has great potentials as a simple supplement applied in the renewable feedstocks-based industrial ABE fermentation at large scale, and the micronutrient zinc-associated response for these selected intracellular metabolisms of *C. acetobutylicum* is significantly pleiotropic, provoking further studies on the systematic mechanism on how zinc acts as an important factor to reprogram metabolic network of *C. acetobutylicum*.

## Methods

### Bacterial culture and fermentation experiment

The strain, pre-culture media, and anaerobical culture condition used in this study were as previously described by Wu *et al.*^30^. The fermentation medium (g/L, carbohydrate 70, yeast extract 2, K_2_HPO_4_ 0.5, KH_2_PO_4_ 0.5, MgSO_4_.7H_2_O 0.2, MnSO_4_.H_2_O 0.01, FeSO_4_.7H_2_O 0.01, CH_3_COONH_4_ 3.22, para-amino-benzoic acid 0.01, biotin 0.01, pH adjusted to 5.5) was supplemented with 0.001 g/L ZnSO_4_.7H_2_O compared to the control. Other than glucose as investigated before, seven other carbohydrates were used as sole carbon source, respectively, including two disaccharides (maltose and cellobiose), three hexoses (galactose, fructose and mannose) and two pentoses (xylose and arabinose). Butanol yield (g/g) was defined as total butanol produced divided by the total carbohydrate utilized; butanol productivity was calculated as total butanol produced divided by the fermentation time and is expressed in g/L/h. During the time course of fermentation using differential carbohydrate as sole carbon source, samples were collected with time intervals of 12 h for cell density and products concentration measurements.

### Analytical methods

Bacterial growth, biomass and extracellular metabolites including acetate, butyrate, acetone, butanol and ethanol were determined as described previously by Wu *et al.*^30^. The differential carbohydrate concentration in fermentation supernatant was determined by 3,5-dinitrosalicylic acid (DNS) method at 540 nm using a spectrophotometer (Thermo Spectronic, USA).

### NAD^+^/NADH assays

Assays were performed with NAD^+^/NADH kit (Sigma, MO) according to the manufacturer’s instructions. Cell pellets were lysed using a Qiagen Tissue Lyser LT (Qiagen, Germany) at 50 oscillations/s for 3 min in the NAD^+^/NADH extraction buffers (Sigma, MO), the resulting lysate was then used to assay for NAD^+^/NADH quantification with the aid of colorimetric indicators at 450 nm. Spectrophotometric measurements were conducted with an iMark^TM^ microplate reader (Bio-Rad, CA).

### LC–MS/MS analysis for intracellular metabolites

All the chemicals used for LC–MS/MS analyses, including standard compounds for intracellular metabolites, were obtained from Sigma-Aldrich. For the preparation of crude extracts, the cells grown in sole glucose medium were collected at 8 h, 16 h, 24 h and 32 h of fermentation respectively by centrifugation at 10000 × g for 3 min at –10 °C, quenched, and extracted rapidly with 500 μL of solution containing methanol, acetonitrile and water (40:40:20, v/v, –40 °C) and then frozen in liquid nitrogen. The samples were then frozen–thawed three times to release metabolites from the cells. The supernatant was collected after centrifugation at 12,000 × g for 3 min at –10 °C. The remaining cell pellets were re-suspended in 500 μL of solution (–40 °C) as described above, and this extraction process was repeated. The supernatant obtained was pooled with that obtained from the first extraction and stored at –80 °C until LC–MS/MS analysis.

LC–MS/MS analysis was conducted on an ACCELA HPLC system (Thermo Scientific, CA) equipped with an ACCELA 1250 pump, an ACCELA auto-sampler and an XBridge BEH Amide column (100 mm × 2.1 mm I.D., 2.5 μm, Waters, Ireland). The mobile phases were 10 mM of ammonium acetate (eluent A) and acetonitrile (eluent B). The gradient program was as follows: from 5% to 60% of eluent A from start to 20 min, isocratic at 60% of eluent A from 20 to 24 min, from 60% to 5% of eluent A from 24 to 25 min. After returning to the initial conditions, the equilibration was achieved after 15 min. Eluent flow rate was set at 200 μL/min and the column temperature was kept at 25 °C. The injection volume was 10 μL with tray temperature of 4 °C. Mass monitoring was achieved using a a TSQ Quantum Ultra triple quadrupole mass analyzer (Thermo Scientific, CA) equipped with a heated electrospray ionization source (HESI). Selective reaction monitoring (SRM) mode was utilized for the monitoring of target compounds. All main working parameters of the mass spectrometer were optimized with flow injection analysis optimization. The main operational parameters of the mass spectrometer are summarized as follows: capillary voltage of 3 KV for positive ionization mode and 2.5 KV for negtive ionization mode, vaporizer temperature of 250 °C, capillary temperature of 300 °C, sheath gas pressure of 35 psi, and aux gas pressure of 10 psi. Xcalibur 2.2 software (Thermo Scientific) was used for instrument control, data acquisition, and processing.

### RNA isolation, cDNA library construction and sequencing

When glucose was used as sole carbon source without/with zinc supplementation, 4 mL of cell cultures at the exponential growth phase of 16 h were collected by centrifuging at 4 °C and 5,000 × g for 5 min prior to suspension in a solution containing RNAprotect reagent and phosphate-buffered saline (PBS) (Qiagen, Germany) with a ratio of 2:1 to stabilize the RNA. The suspension was incubated at room temperature for 5 min to obtain cell pellets by centrifuging, which were then immediately shock-frozen in liquid nitrogen and stored at –80 °C for further RNA isolation. The target total cellular RNA was purified using the RNeasy Mini Kit (Qiagen, Germany) according to the manufacturer’s instructions. RNA quality and integrity were analyzed using a Nanochip 2130 bioanalyzer (Agilent Technologies, USA), and the RNA integrity number (RIN) of each sample used for RNA-Seq was no less than 7.0. The cDNA libraries construction and sequencing were both performed by the Beijing Genomics Institute (BGI, Shenzhen, China). The final cDNA libraries were further qualified and quantified using 2130 Bioanaylzer (Agilent Technologies, USA) and StepOnePlus Real-Time PCR System (Applied Biosystems) followed by sequencing via the HiSeq 2000 system (Illumina).

### RNA-Seq data analysis

The generated 100-nt reads via single-end technology were mapped to the *C. acetobutylicum* ATCC 824 genome, and those that did not align uniquely with the genome were discarded. The levels of the differentially expressed genes (DEGs) were normalized by RPKM (reads/Kb/Million), defined as the number of reads per kilobase of the exon region per million mapped reads. FDR (false discovery rate) was used to determine the threshold of the p-value for this analysis. Furthermore, in this study, the Log2 ratio of RPKM between samples was used to calculate the fold-change values. To screen the DEGs between samples, we used a FDR of ≤0.001 as the threshold to judge the significance of differential gene expression.

### Quantitative real-time PCR (Q-RT-PCR)

Quantitative real-time PCR (Q-RT-PCR) was performed for selected genes at different sampling times with 0.0005 and 0.001 g/L ZnSO_4_.7H_2_O supplementation in order to validate the relative quantification and dynamic changes of key genes expression. Eleven genes involved in carbohydrate utilization, glycolysis, acidogenesis and solventogenesis and their specific primer sequences are listed in Table 2. The gene CAC0905 encoding NAD(FAD)-dependent dehydrogenase in *C. acetobutylicum* was used as the housekeeping gene based on its relatively constant expression level under the tested condition. Triplicate reactions were performed using SYBR® Premix Ex TaqTM II (Takara Bio Inc.) for supporting the validity of the RNA-seq data.

## Additional Information

**How to cite this article**: Wu, Y.-D. *et al.* Transcriptional analysis of micronutrient zinc-associated response for enhanced carbohydrate utilization and earlier solventogenesis in *Clostridium acetobutylicum. Sci. Rep.* **5**, 16598; doi: 10.1038/srep16598 (2015).

## Supplementary Material

Supplementary Information

## Figures and Tables

**Table 1:** Performances of batch ABE fermentations by *C. acetobutylicum* using differential carbohydrate (without/ with zinc supplementation).

Carbonsource	Carbohydrateutilization (g/L)	Max. OD_620_	Products (g/L)					Productivity_B_(g/L/h)
			Acetate	Butyrate	Acetone	Butanol	Ethanol	
Maltose	58.0 ± 1.2/65.5 ± 1.2	3.8 ± 0.2/4.1 ± 0.2	2.9 ± 0.2/2.6 ± 0.2	1.5 ± 0.2/1.0 ± 0.2	8.8 ± 0.4/9.8 ± 0.6	13.7 ± 0.6/15.9 ± 0.8	2.2 ± 0.1/2.4 ± 0.1	0.190/0.221
Cellobiose	56.4 ± 1.4/58.5 ± 1.1	3.1 ± 0.1/3.3 ± 0.1	2.5 ± 0.1/2.2 ± 0.1	1.1 ± 0.1/0.9 ± 0.1	7.9 ± 0.4/8.3 ± 0.5	11.4 ± 0.5/12.4 ± 0.5	1.5 ± 0.1/1.6 ± 0.1	0.095/0.103
Glucose	55.4 ± 1.8/55.0 ± 0.8	4.2 ± 0.1/5.2 ± 0.2	0.9 ± 0.1/0.5 ± 0.1	0.8 ± 0.1/0.7 ± 0.1	6.4 ± 0.2/6.7 ± 0.4	11.7 ± 0.4/12.6 ± 0.3	1.3 ± 0.1/1.7 ± 0.1	0.183/0.315
Fructose	43.6 ± 1.1/63.5 ± 1.6	1.7 ± 0.1/1.9 ± 0.1	2.7 ± 0.1/1.5 ± 0.1	2.4 ± 0.1/0.5 ± 0.1	2.8 ± 0.2/7.3 ± 0.4	4.5 ± 0.3/12.8 ± 0.5	0.3 ± 0.0/1.0 ± 0.1	0.028/0.089
Galactose	15.4 ± 0.6/15.2 ± 0.5	2.0 ± 0.1/2.1 ± 0.1	8.0 ± 0.4/7.7 ± 0.4	4.1 ± 0.2/3.9 ± 0.2	0.3 ± 0.0/0.3 ± 0.0	0.4 ± 0.0/0.4 ± 0.0	0.2 ± 0.0/0.2 ± 0.0	0.005/0.006
Mannose	17.0 ± 0.4/16.4 ± 0.5	2.3 ± 0.1/2.1 ± 0.1	3.1 ± 0.2/3.4 ± 0.2	1.4 ± 0.1/1.5 ± 0.1	0.8 ± 0.1/0.7 ± 0.1	1.0 ± 0.1/0.8 ± 0.1	0.1 ± 0.0/0.1 ± 0.0	0.027/0.013
Xylose	38.1 ± 1.0/45.2 ± 1.3	2.4 ± 0.2/2.8 ± 0.2	1.7 ± 0.1/1.3 ± 0.1	3.0 ± 0.2/2.0 ± 0.1	4.4 ± 0.2/5.2 ± 0.3	6.3 ± 0.3/8.3 ± 0.4	0.4 ± 0.0/0.5 ± 0.0	0.075/0.099
Arabinose	60.0 ± 1.6/62.4 ± 1.4	3.5 ± 0.2/3.8 ± 0.2	3.5 ± 0.2/3.1 ± 0.2	1.5 ± 0.1/1.1 ± 0.1	8.7 ± 0.4/9.2 ± 0.5	9.9 ± 0.5/10.8 ± 0.5	1.6 ± 0.1/1.7 ± 0.1	0.069/0.090

**Table 2:** Q-RT-PCR verification for genes expression at different times with 0.0005 and 0.001 g/L ZnSO_4_.7H_2_O supplementation.

Gene	Forward primer sequence (5′–3′)	Reverse primer sequence (5′–3′)	RNA-Seq	Q-RT-PCR (Zinc/Control)			
				8 h(0.001)	16 h(0.001)	32 h(0.001)	16 h(0.0005)
CAC0234	CAATGGCAGCAGGAATGGT	TGGTATTGCGCCTTCTGTTATG	2.76	3.27	4.03	0.78	4.16
CAC0386	CAAACTTCCAGAAAGCGTACCA	AACACCAAACCACCAGAAGAAAG	5.19	5.96	7.78	0.66	6.33
CAC0570	AAATGCTTGGAGACGGTTTTG	AGTCCGCCTTCTGTTGTTATTG	3.62	4.93	4.98	0.53	3.82
CAC1353	GCACTTCCAGCTGCATGTTTAG	TCCAAGAAGCATACCAGAAACCT	1.76	1.46	2.01	0.46	2.05
CAC1354	AAGTAGGGCAGCCGATTATAAAAGT	TGGTTTCCAGCGTCAACATC	1.77	1.94	2.27	0.36	2.33
CAC1342	GGAATGAAGCAATTACCTGGACTT	GCTGTTTTCCAATCGCCTTC	4.84	3.33	6.07	0.59	5.74
CAC2613	ATTTCTCTCAAAGAGGAAGGTTCAC	TTTGTGGATGTGGAAAGACAGG	1.21	1.55	1.89	0.72	1.47
CAC0078	GGTTCTGGCGTCTTTTATAATCGT	TTTAGCTTGTCCCTCAGTTTAGCTC	0.65	0.70	0.66	0.83	0.79
CAC2873	ACAGTTTCTCCCTTTCTGCCTTT	AAGATGAGTTTGCTCTTGCATCAC	1.46	1.42	2.45	0.67	1.97
CAC3298	CCCTACGCCGTGTGTTATGTC	TGAGGCAAGAGCCAATCTAATGT	1.35	1.91	2.57	0.65	2.28
CAP0164	TTCGATAGCTCAGTTTCGTTTTCAC	CCACCCATACCAGAGAGCATTT	1.62	1.78	2.93	0.75	2.06
CAC0905	TGGATTACAAGCAGCCATTTC	CTGTTCCCGTAAGCTCCTCTTT	1.00	Housekeeping gene			
