# Supplementary material for: Transcriptional analysis of micronutrient zinc-associated response for enhanced carbohydrate utilization and earlier solventogenesis in Clostridium acetobutylicum
Source: Sci Rep. 2015 Nov 20;5:16598. doi: 10.1038/srep16598 (PMC4653742; doi:10.1038/srep16598)
Supplement: Supplementary Information [file srep16598-s1.doc]

**Transcriptional analysis of micronutrient zinc**-**associated response for enhanced carbohydrate utilization and earlier solventogenesis in *Clostridium acetobutylicum***

You-Duo Wu^a^, Chuang Xue^a^, Li-Jie Chena[[1]](#footnote-2)*, Hui-Hui Wan^b^, Feng-Wu Bai^a,c^

*^a^School of Life Science and Biotechnology, Dalian University of Technology, Dalian 116024, China*

*^b^State Key Laboratory of Fine Chemicals, Dalian University of Technology, Dalian 116024, China*

*^c^School of Life Sciences and Biotechnology, Shanghai Jiao Tong University, Shanghai 200240, China*

**Emails:** duoduotheone@mail.dlut.edu.cn (Y. D. Wu)

[xue.1@dlut.edu.cn](mailto:xue.1@dlut.edu.cn) (C. Xue)

[ljchen@dlut.edu.cn](mailto:ljchen@dlut.edu.cn) (L. J. Chen)

[wanhuihui@dlut.edu.cn](mailto:wanhuihui@dlut.edu.cn) (H. H. W)

[fwbai@sjtu.edu.cn](mailto:fwbai@sjtu.edu.cn) (F. W. Bai)

Table S1 Comparison of influenced transcripts involved in differential carbohydrate utilization of *C. acetobutylicum* without/with supplementary zinc

| ORF No. | Gene name | Protein function | Expression ratio (Zinc/Control) |
| --- | --- | --- | --- |
| Polysaccharide and disaccharide metabolic genes | | | |
| CAC0332 | *manB* | Beta-mannanase | 2.11 |
| CAC0383 |  | Cellobiose-specific PTS system transporter subunit IIA | 3.20 |
| CAC0384 | *licB* | Cellobiose-specific PTS system transporter subunit IIB | 3.84 |
| CAC0385 |  | Beta-glucosidase | 4.22 |
| CAC0386 | *licC* | Cellobiose-specific PTS system transporter subunit IIC | 5.19 |
| CAC0532 | *malP* | Arbutin-like PTS system transporter subunit IIBC | 2.66 |
| CAC0533 | *malH* | Maltose-6’-phosphate glucosidase | 1.51 |
| CAC0706 |  | Endo-1,4-beta glucanase | 1.56 |
| CAC1075 |  | Beta-glucosidase | 1.81 |
| CAC1084 |  | Beta-glucosidase | 2.25 |
| CAC2252 |  | Alpha-glucosidase | 2.87 |
| CAC2807 |  | Endo-1,3(4)-beta-glucanase 16 | 1.53 |
| CAC2810 |  | Glucoamylase | 2.03 |
| CAC2891 |  | Bifunctional alpha-glucosidase/glycosidase | 1.79 |
| Hexose metabolic genes | | | |
| CAP0066 | *ptnA* | Mannose-specific PTS system transporter subunit IIAB | 0.45 |
| CAP0067 | *manY/levF* | ABC transporter permease | 0.34 |
| CAP0068 | *ptnD* | ABC transporter permease | 0.33 |
| CAC0231 | *fruR* | Putative DeoR-type transcriptional regulator | 1.51 |
| CAC0232 | *fruB* | 1-Phosphofructokinase | 1.97 |
| CAC0233 | *fruC* | PTS system transporter subunit IIA | 2.59 |
| CAC0234 | *fruD* | fructoso-specific PTS system transporter subunit IIBC; | 2.76 |
| CAC0570 | *glcG* | PTS system transporter subunit IICBA | 3.62 |
| CAC1353 |  | PTS system transporter subunit IICB | 1.76 |

Table S1 (Continued)

| ORF No. | Gene name | Protein function | Expression ratio (Zinc/Control) |
| --- | --- | --- | --- |
| CAC1354 |  | PTS system transporter subunit IIA | 1.77 |
| CAC2237 | *glgC* | Glucose-1-phosphate adenylyltransferase | 1.88 |
| CAC2238 | *glgC* | ADP-glucose pyrophosphorylase | 2.05 |
| CAC2239 | *glgA* | Glycogen synthase | 1.84 |
| Pentose metabolic genes | | | |
| CAC1341 | *araD* | L-ribulose-5-phosphate 4-epimerase | 2.33 |
| CAC1342 | *araA* | L-arabinose isomerase | 4.84 |
| CAC1347 | *tal* | Transaldolase | 3.19 |
| CAC1348 | *tkt* | Transketolase | 2.44 |
| CAC1349 | *galM* | Aldolase-3-epimerase | 2.77 |
| CAC1730 | *rpe* | Ribulose-phosphate 3-epimerase | 1.52 |
| CAC2610 | *xylA* | Xylose isomerase | 2.29 |
| CAC2612 | *xylB* | Xylulose kinase | 2.31 |

Table S2 Comparative analysis of genes responsible for glycolysis, acidogenesis and solventogenesis of *C. acetobutylicum* without/with supplementary zinc

| ORF No. | Gene name | Protein function | Expression ratio (Zinc/Control) |
| --- | --- | --- | --- |
| Glycolytic genes | | | |
| CAC0028 | *hydA* | Hydrogene dehydrogenase | 0.86 |
| CAC0517 | *pfkA* | 6-Phosphofructokinase | 1.01 |
| CAC0518 | *pykA* | Pyruvate kinase | 1.16 |
| CAC0709 | *gapC* | Glyceraldehyde 3-P dehydrogenase | 1.01 |
| CAC0710 | *pgK* | Phosphoglycerate kinase | 1.16 |
| CAC0711 | *tgi* | Triosephosphate isomerase | 1.14 |
| CAC0712 | *pgm* | Phosphoglycero mutase | 1.17 |
| CAC0713 | *eno* | Phosphopyruvate hydratase | 1.15 |
| CAC0827 | *fba* | Fructose-bisphosphate aldolase | 1.01 |
| CAC1036 | *pykA* | Pyruvate kinase | 1.19 |
| CAC2613 | *glcK* | Putative glucokinase | 1.21 |
| CAC2680 | *pgi* | Glucose-6-phosphate isomerase | 1.07 |
| Acidogenic genes | | | |
| CAC0078 | *thlB* | Acetyl-CoA acetyltransferase | 0.65 |
| CAC1742 | *pta* | Phosphate acetyltransferase | 1.04 |
| CAC1743 | *ack* | Acetate kinase | 0.95 |
| CAC2708 | *hbd* | β-hydroxybutyryl-CoA dehydrogenase | 0.96 |
| CAC2709 | *etfA* | Electron transfer flavoprotein | 1.33 |
| CAC2710 | *etfB* | Electron transfer flavoprotein | 1.36 |
| CAC2711 | *bcd* | Butyryl-CoA dehydrogenase | 1.31 |
| CAC2712 | *crt* | 3-hydroxybutyryl-CoA dehydratase | 1.72 |
| CAC2873 | *thlA* | Thiolase | 1.46 |
| CAC3075 | *buk* | Butyrate kinase | 0.97 |
| CAC3076 | *ptb* | Phosphate butyryltransferase | 0.97 |

Table S2 (Continued)

| ORF No. | Gene name | Protein function | Expression ratio (Zinc/Control) |
| --- | --- | --- | --- |
| Solventogenic genes | | | |
| CAP0035 | *adhE2* | Aldehyde alcohol dehydrogenase | 1.01 |
| CAP0059 |  | Hypothetical protein | 0.21 |
| CAP0162 | *aad* | Aldehyde alcohol dehydrogenase | 0.61 |
| CAP0163 | *ctfA* | CoA-transferase A | N.D. |
| CAP0164 | *ctfB* | CoA-transferase B | 1.62 |
| CAP0165 | *adc* | Acetoacetate decarboxylase | 1.41 |
| CAC3292 |  | NifU-like protein involved in Fe-S cluster formation | 0.63 |
| CAC3298 | *bdhB* | Butanol dehydrogenase B | 1.35 |
| CAC3299 | *bdhA* | Butanol dehydrogenase A | 1.08 |

1. ^*^ Correspondence: Tel & Fax: 86-0411-8470 6308; E-mail: [ljchen@dlut.edu.cn](mailto:ljchen@dlut.edu.cn) (L.J. Chen) [↑](#footnote-ref-2)
